# Interpretation of Positive Roche Elecsys SARS-CoV-2 Antigen Test Results: Value for Clinical Decisions

**DOI:** 10.1155/cjid/9054868

**Published:** 2025-11-21

**Authors:** Shinsuke Mizuno, Yoshiyuki Tsukamura, Masashi Kasai

**Affiliations:** ^1^Division of Infectious Disease, Department of Pediatrics, Hyogo Prefectural Kobe Children's Hospital, Kobe, Hyogo, Japan; ^2^Department of Inspection Unit, Hyogo Prefectural Kobe Children's Hospital, Kobe, Hyogo, Japan

**Keywords:** coronavirus disease 19, false-positive result, quantitative antigen test, reverse transcription-polymerase chain reaction test, screening test

## Abstract

The Elecsys SARS-CoV-2 antigen test (Elecsys; Roche Diagnostics, Rotkreuz, Switzerland) is a quantitative test for detecting severe acute respiratory syndrome coronavirus 2 (SARS-CoV-2) antigen. Although the positive predictive value of the test is high, false-positive (FP) results are unavoidable. Furthermore, FP results can lead to unnecessary isolation of patients or exposure of FP patients to truly positive patients. Therefore, to minimize potential harm to FP patients, this study aimed to identify clinical cases that are likely to produce FP results and in which additional testing should be recommended. These recommendations should be based on the Elecsys test results and relevant clinical information. Correspondingly, a retrospective study was conducted using nasopharyngeal swab samples from symptomatic and asymptomatic patients with SARS-CoV-2 infection and patients who had close contact with a confirmed SARS-CoV-2 infection case between September 2021 and October 2023. Of the total 5989 samples, 302 tested positive in the Elecsys test. Thereafter, the reverse transcription-polymerase chain reaction test was performed on patient samples (*n* = 54) with positive Elecsys test results without clinical information consistent with coronavirus disease 19 (COVID-19). A total of 37 of these samples were FP results, and all FP samples showed Elecsys test values near the cutoff index (COI) value. Accordingly, when the Elecsys test yields a low COI value, and the clinical presentation is inconsistent with COVID-19, additional confirmatory testing is recommended.

## 1. Introduction

Although reverse transcription-polymerase chain reaction (RT-PCR) testing remains the gold standard for the diagnosis of coronavirus disease 2019 (COVID-19), several antigen tests have been introduced in recent years as faster, simpler, and more cost-effective alternatives. Among these antigen tests, quantitative assays demonstrate higher sensitivity and specificity than qualitative tests and are recognized as reliable diagnostic tools in Japan. Nine quantitative antigen tests, including Lumipulse Presto SARS-CoV-2 Antigen (Lumipulse; Fujirebio, Tokyo, Japan) and Elecsys SARS-CoV-2 Antigen (Elecsys; Roche Diagnostics, Rotkreuz, Switzerland), have been approved in Japan [1]. The Elecsys test is based on an electrochemiluminescence immunoassay that applies the antibody sandwich principle. Owing to its high specificity, the manufacturer has not provided recommendations for additional RT-PCR testing of positive samples [2]. Moreover, the Japanese Ministry of Health, Labor, and Welfare permits a definitive COVID-19 diagnosis using positive antigen test results without further confirmation via nucleic acid amplification tests. However, immunoassay approaches can produce false-positive (FP) results, and clinical data regarding the FP rates of the Elecsys test and clinical information associated with FP cases are limited [3].

FP results cause unnecessary isolation of patients, which exposes them to patients with COVID-19, and subsequent nosocomial severe acute respiratory syndrome coronavirus 2 (SARS-CoV-2) infection. This delays comorbidity treatment, induces physical and mental stress, and increases the burden on medical staff and facilities [4]. Accordingly, this study aimed to evaluate the frequency and test characteristics of FP cases based on the Elecsys test results and clinical information. Furthermore, the study aimed to identify criteria for recommending additional PCR testing.

## 2. Materials and Methods

### 2.1. Study Design

This retrospective cohort study included Elecsys test results of all patients hospitalized at Hyogo Prefectural Kobe Children's Hospital between September 1, 2021, and October 31, 2023.

### 2.2. Sample Collection

Nasopharyngeal samples were collected from 5989 individuals admitted to the emergency room or clinical wards. Samples were collected from patients with symptoms consistent with COVID-19, patients who had been in contact with confirmed COVID-19 cases on admission or during hospitalization, and patients who were screened upon admission from the emergency department or for prevention of nosocomial infection during hospitalization. Patients admitted with symptoms consistent with COVID-19 but with a clearly documented alternative reason were classified as having an asymptomatic diagnosis. Samples were collected using nylon-flocked swabs and placed in sterile tubes. The extraction solution included in the Elecsys test kit (JAN: 4987518316938) was added to each tube, and each sample with extraction solution was stored within 2 days at 2°C–8°C according to the product instructions. Samples that required further RT-PCR testing were preserved at −80°C after antigen testing.

### 2.3. Definitions

Symptoms consistent with COVID-19 were defined as documented fever, respiratory symptoms (cough, shortness of breath, or difficulty breathing), gastrointestinal symptoms (nausea, vomiting, or abdominal pain), or neurological symptoms (seizure, alteration of consciousness, dysgeusia, or olfactory dysfunction) before or during hospitalization. FP results were defined as samples that yielded a positive Elecsys SARS-CoV-2 antigen result (cutoff index [COI] ≥ 1.0) but tested negative in the confirmatory RT-PCR tests using either the Cobas Liat SARS-CoV-2 & Flu A/B (Liat; Roche Diagnostics) or BioFire FilmArray Respiratory Panel (FilmArray; BioFire Diagnostics, Salt Lake City, UT, USA) platforms.

### 2.4. SARS-CoV-2 Antigen Detection

SARS-CoV-2 antigen was detected using the Elecsys test fully automated Cobas e 411 analyzer according to the manufacturer's instructions [5]. Positive or negative results were determined based on the antigen COI values (negative: COI < 1.0 and positive: COI ≥ 1.0).

### 2.5. SARS-CoV-2 Detection via RT-PCR

The SARS-CoV-2 RT-PCR test was performed using the Liat and FilmArray tests, according to the manufacturer's instructions [6]. These tests were used in the study hospital and incorporated as RT-PCR tests in this study. RT-PCR confirmation was performed on samples with COI values between 1.0 and 10.0 with the absence of known COVID-19 exposure or when clinical findings were inconsistent with COVID-19 (lack of fever or respiratory, gastrointestinal, or neurological symptoms). To avoid viral replication, each sample was recollected from the patient within 30 min of first collection, or the residual test buffer (RTB) from a positive antigen test was used. Variations near the cutoff are expected, and slight deviations from the kit composition or manufacturer's instructions can result in FPs. Therefore, all suspected FP specimens were retested by different laboratory technicians to confirm concordance of results. All assays were performed according to the manufacturer's protocol. The test results were considered positive if the FilmArray test was positive for SARS-CoV-2 or if the Liat test had a cycle threshold < 35; the tests were otherwise judged as negative. Any samples with a positive Elecsys test and negative RT-PCR test were considered FPs.

### 2.6. Quality Management

In accordance with ISO 15189, to control the accuracy of the Elecsys test, the precontrol SARS-CoV-2 antigen was measured twice a day to confirm that it was within the reference range. Shift trends were checked using control measurement data collected over time. For the FilmArray test, controls were not measured separately, and for the Liat test, controls were only measured at lot changes. For the FilmArray and Liat test, an internal control was automatically analyzed for each measurement. If the result of the internal control was invalid, the result was not released.

## 3. Results

In total, 302 (5.0%) of the 5989 nasopharyngeal swab samples tested positive in the Elecsys test. Of these, 54 samples were collected from patients who showed no clinical symptoms consistent with COVID-19, and additional RT-PCR tests revealed that 37 of these samples were FPs. Furthermore, 269 samples tested negative in the Elecsys test, and these patients showed clinical symptoms consistent with COVID-19. Therefore, an additional RT-PCR was performed for these samples, and seven showed positive RT-PCR results. Four of these samples were taken from patients with a history of COVID-19 up to two months prior to the examination, and three of these samples were from patients with symptoms consistent with COVID-19 who had close contact with other patients with COVID-19. Based on these results, the sensitivity was estimated as 97.4% (95% confidence interval [CI]: 95.5%–99.4%) and the specificity was estimated as 99.4% (95% CI: 99.1%–99.6%).

**Figure 1:**
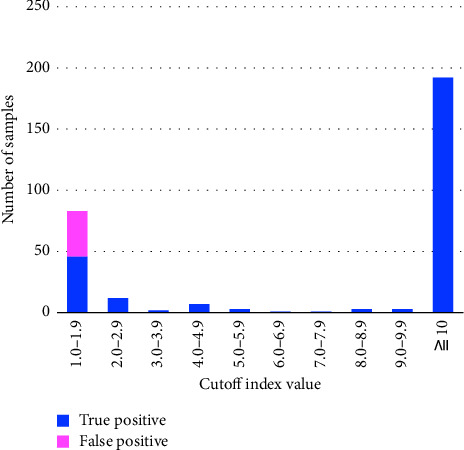
Distribution of Elecsys SARS-CoV-2 antigen test COI values during the delta- and omicron-predominant periods. When the antigen level was low (COI threshold = 1.0–10.0) and the physician suspected a FP because of the clinical context (fever, respiratory symptoms, gastrointestinal symptoms, neurological symptoms, or sick patient contact), an additional RT-PCR was performed. FP, false positive; TP, true positives; SARS-CoV-2, severe acute respiratory syndrome coronavirus 2; COI, cutoff index; RT-PCR, reverse transcription-polymerase chain reaction.

The clinical characteristics of the FP patients are shown in Table 1. Approximately 64.9% (24/37) of patients had underlying diseases and 48.6% (18/37) of patients had symptoms consistent with COVID-19. Of these 18 samples, four samples were positive for other respiratory pathogens as identified by the FilmArray test, and three samples were diagnosed as bacterial pneumonia. Moreover, four were diagnosed as acute abdominal diseases (two acute appendicitis, one acute cholangitis, and one ovarian torsion). Two of the patients had brain tumors, and two exhibited drug side effects. Only two patients had contact with confirmed COVID-19 cases; however, no symptoms were observed during the follow-up. The distribution of the COI values for the Elecsys tests is shown in Figure 1. Among the 37 FP samples, the median COI value was 1.12 (interquartile range: 1.05–1.18), and 91.9% were below 1.3 COI. All patients were moved to the infectious disease ward, and five were treated for COVID-19.

## 4. Discussion

In this study, 37 of the 5989 samples showed FP results, and the antigen levels were within the COI range of 1.01–1.28. None of the FP patients had both symptoms consistent with COVID-19 and a history of contact with confirmed COVID-19 cases. These findings suggest that the Elecsys test has the potential to produce FP results in specific cases, particularly when COI values are low and the clinical context is inconsistent with COVID-19. This therefore highlights the importance of confirmatory testing for these cases.

These study findings are consistent with those of Osterman et al. [7], who reported an FP rate of 2.3% (7/303 samples) for the Elecsys test. Similarly, Montalvo et al. [8] observed more frequent FPs (6.2%) in the Elecsys test. The Lumipulse antigen test, which is widely used in Japan, requires a confirmation RT-PCR test when the quantitative value is intermediate. Yokota et al. [9] observed that screening tests for asymptomatic individuals yielded intermediate results in 513/88,924 samples, and confirmatory tests were negative in 93.4% of these cases. Based on these findings, it is recommended that clinicians interpret Elecsys results cautiously when the COI is < 2.0 and the clinical picture is inconsistent with COVID-19. Moreover, it is recommended that confirmatory RT-PCR be performed before isolation or treatment decisions.

The FP rate varies according to estimated prevalence; therefore, caution is required when making clinical decisions based solely on this value. For example, based on the findings of this study, when the prevalence is 1%, the FP rate is 39.8%. However, at a prevalence of 10%, the FP rate decreases to 5.7%. The high prevalence period is defined as the duration of the following five COVID-19 epidemic waves in Japan: September 2021, January to March 2022, July to September 2022, November 2022 to January 2023, and July to September 2023 [10]. The low prevalence period is defined as any other period. This study was conducted during the period in which Omicron was the predominant strain, and the epidemic continued to be prevalent outside of an epidemic wave period [10]. The prevalence among children, young adults, and pregnant women was relatively high during this period. Nevertheless, the frequency of FP results was not negligible.

Considering that the immunoassay method detects the reactivity of the antigen with the antibody, FP results may have been caused by nonspecific reactions, such as cross-reactions with other viruses. Montalvo et al. [9] did not observe cross-reactivity with other respiratory viruses. Hence, other possible mechanisms for the FP in this study include high specimen viscosity and interference of human antibodies. However, these mechanisms are difficult to validate.

This study had some limitations. First, as this was a retrospective study from a tertiary medical center, the study findings cannot be generalized to other medical facilities. However, FP Elecsys test results have been observed in adult and mixed populations [8, 9]. Therefore, physicians should not limit FP Elecsys test results to pediatric and pregnant populations but should also consider FP results in mixed populations. Second, because RT-PCR was only performed for samples suspected of FP results based on clinical context, verification bias could not be excluded. This selective confirmation may lead to an overestimation of the FP rate. Furthermore, considering that not all positive results were recorded, reporting of false detection rates is also not possible. Although reporting FP rates from a subset of preselected results artificially increases the value, the estimated FP rate was not negligible. Third, the study data were obtained during the Omicron-predominant period. The Elecsys test is based on a monoclonal antibody, and the Elecsys antibody is expected to react with conserved domains of the nucleocapsid protein. Therefore, other variant strains can also be detected in the same manner; however, further investigation is needed to account for future variants. Fourth, the RTB from the Elecsys test kit was used for RT-PCR. Although several samples with low COI values tested positive in the RT-PCR test, the lack of interference of the pretreatment solution in the PCR was not confirmed. However, most pretreatment solutions are designed to inactivate viruses and are unlikely to interfere with nucleic acid detection [11, 12]. Nonetheless, as the composition of the pretreatment solution used in this study is not disclosed, further validation studies are needed to ensure that it does not inhibit amplification.

## 5. Conclusions

**Figure 2:**
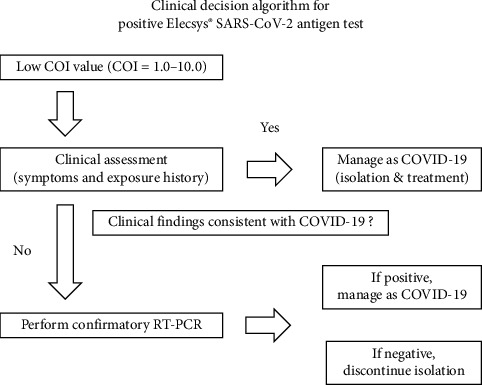
Flowchart of the clinical decision algorithm for low COI values. COI, cutoff index; COVID-19, coronavirus disease 2019; RT-PCR, reverse transcription-polymerase chain reaction; SARS-CoV-2, severe acute respiratory syndrome coronavirus 2.

Although the Elecsys antigen test demonstrates high specificity, FP results can occur at low COI values. Therefore, an additional RT-PCR confirmation is recommended for samples with COI values of < 2.0 when clinical findings do not support COVID-19 (Figure 2). Accordingly, establishing institution-specific COI thresholds through prospective validation will further optimize diagnostic accuracy and patient safety.

## Figures and Tables

**Table 1:** Clinical information among false-positive Elecsys SARS-CoV-2 antigen test results.

**Variables**	

Age (median year (IQR))	10 (5–18)
Sex (male *n* (%))	16 (43.2%)
Underlying disease or condition (*n* (%))	
None	13 (35.1%)
Blood cancer and oncologic disease	7 (18.9%)
Chronic heart disease	6 (16.2%)
Neurological and neuromuscular disease	5 (13.5%)
Pregnancy	5 (13.5%)
Chronic pulmonary disease	4 (10.8%)
Others	8 (21.6%)
Symptoms (*n* (%))	
None	19 (51.4%)
Fever	10 (27.0%)
Respiratory symptoms	8 (21.6%)
Gastrointestinal symptoms	6 (16.2%)
Antigen level (median COI (IQR))	1.07 (1.03–1.15)

*Note: n*, number of individuals.

Abbreviations: COI = cutoff index, IQR = interquartile range.

## Data Availability

The datasets generated and/or analyzed during this study are available from the corresponding author on reasonable request.
